# Milk Fat Globule Epidermal Growth Factor 8 (MFGE8) Gene Variants in Rheumatoid Arthritis and Sjögren’s Syndrome

**DOI:** 10.3390/jcm11051180

**Published:** 2022-02-23

**Authors:** Charalampos Skarlis, Adrianos Nezos, Antonios Chatzigeorgiou, Clio P. Mavragani

**Affiliations:** 1 Department of Physiology, School of Medicine, National and Kapodistrian University of Athens, 11527 Athens, Greece; charskarlis@med.uoa.gr (C.S.); anezos@med.uoa.gr (A.N.); achatzig@med.uoa.gr (A.C.); 2 Joint Academic Rheumatology Program, School of Medicine, National and Kapodistrian University of Athens, 11527 Athens, Greece

**Keywords:** MFGE8, systemic lupus erythematosus, rheumatoid arthritis, Sjögren’s Syndrome, polymorphisms

## Abstract

Milk Fat Globule Epidermal Growth Factor 8 (MFGE8) deficiency and gene polymorphisms have been previously linked to systemic lupus erythematosus (SLE)-like and SLE development. Our aim was to explore whether four MFGE8 variants and MFGE8 serum levels are associated with autoimmunity susceptibility and autoimmune related atherosclerosis. DNA from 107 primary Sjogren’s syndrome (SS), 116 rheumatoid arthritis (RA) and 123 SLE patients as well as 199 HC were genotyped for the MFGE8 rs2271715, rs1878326, rs4945, rs3743388 variants by RFLP-PCR. MFGE8 serum levels were measured by ELISA. The CA genotype of rs4945 variant exhibited a protective effect against RA development, a finding not confirmed in the SS and SLE populations. The CACG haplotype exhibited a protective effect in both RA and SS patients compared to HC. Primary SS patients with IMT ≤ 0.9 mm displayed higher MGFE8 serum levels compared to those with ˃0.9 mm. Here, we report a novel association of MFGE8 variants in SS and RA susceptibility, as well as reduced MFGE8 serum levels in SS patients with heightened atherosclerotic risk.

## 1. Introduction

Milk fat globule epidermal growth factor 8 (MFGE8) is a secreted integrin-binding protein, found in mammary epithelial cell surfaces and aortic media. Physiologically, MFGE8 binds to phosphatidylserine at the surface of apoptotic cells and enables their recognition by ανβ3/β5 integrins, promoting the phagocytosis of apoptotic cells by macrophages and the development of subsequent tolerogenic immune responses. Of interest, MFGE8 deficiency in mice delays the clearance of apoptotic lymphocytes, leading to enhanced self-antigen presentation and generation of a disease reminiscent of human systemic lupus erythematosus (SLE) [1].

Previous studies suggest that MFGE8 serum levels are associated with severe atherosclerosis, acting as an age-related proinflammatory factor [2]. Additionally, MFGE8 gene polymorphisms have been associated with autoimmunity development in humans. A Korean study reported that SLE patients carrying the MFGE8 rs2271715 CC and rs4945 CA or AA genotypes display higher MFGE8 serum levels, while the rs2271715 and rs3743388 polymorphism a associated with increased risk for SLE development [1]. 

**Figure 1:**
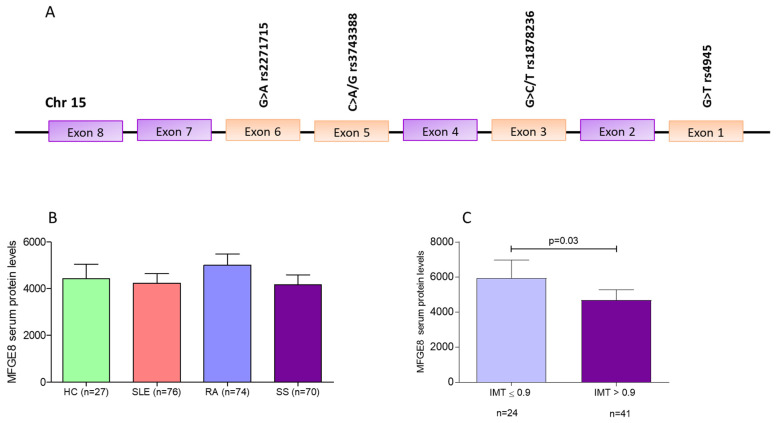
Chromosomal location of the MFGE8 variants and MFGE8 protein serum levels between SLE, RA, SS patients and HC. (**A**). Chromosomal location of the MFGE8 variants (**B**). No differences in MFGE8 serum levels were found between SLE, RA, SS patients and HC. (**C**). Significantly higher MFGE8 protein levels in the serum of SS patients with IMT ≤ 0.9 mm compared to SS patients with IMT > 0.9 mm. MFGE8: Milk fat globule epidermal growth factor 8; IMT: intima media thickness; RA: Rheumatoid Arthritis; SS: Sjögren’s Syndrome; SLE: Systemic Lupus Erythematosus; HC: healthy controls; *p* = 0.05.

Given that patients suffering by systemic autoimmune diseases display an excessive burden for subclinical atherosclerosis [3,4,5,6], the aim of our study was to examine whether four MFGE8 variants—previously linked to autoimmunity development in a Taiwanese study—(Figure 1A) and MFGE8 serum levels are associated with autoimmune disease susceptibility, as well as autoimmune-related atherosclerosis in Caucasian patients [7].

## 2. Patients and Methods

In the present case-control study, DNA derived from peripheral blood of 107 Sjögren’s Syndrome (SS), 116 Rheumatoid arthritis (RA), 123 SLE patients and 199 HC, was extracted, stored and implemented for genotyping studies at the Molecular and Applied Physiology Unit, Department of Physiology, National and Kapodistrian University of Athens. All SS, SLE and RA patients fulfilled the 2016 SS [8], 2019 SLE [9] and 2010 RA [10] classification criteria, respectively. Patients younger than 18 years were excluded from the study. Clinical, serological, histopathological characteristics, as well as markers for subclinical atherosclerosis (carotid plaque and arterial wall thickening, defined as intima media thickness (IMT) scores more than 0.90 mm) were collected for all patients, as previously described [4,5]. All study participants provided written informed consent according to the Declaration of Helsinki.

## 3. Restriction Fragment Length Polymorphism-Polymerase Chain Reaction (RFLP-PCR)

Patients and HC were genotyped for the MFGE8 rs2271715, rs1878326, rs4945, rs3743388 single nucleotide polymorphisms by restriction fragment length polymorphism (RFLP-PCR). Briefly, following the DNA extraction from whole peripheral blood, a genomic DNA aliquot (30–50 ng/mL) was amplified by polymerase chain reaction (PCR), using the following primers: for rs2271715 F: 5′-AAG GTA GGT CCT GTT GGG GC-3′ and R: 5′-GGC TCA GAA TGA AAC CCA G**C**-3′, for rs1878326 F: 5′-GGC TTA GG**C** AGA GGT CAT ACA G-3′ and R: 5′-CTG CAA ACC CAA GAA GGT CAC-3′, for rs4945 F: 5′-GTG CTG AGC CGC CTG ATT TA-3′ and R: 5′-TCA CCC AGG GCG ACG A-3′, and for rs3743388 F: 5′-GGT TTA TGC CCA GGC TCC AT-3′ and R: 5′-TCT CAC GTA CTG AGC CTC CA-3′. PCR amplification for all SNPs was performed in a Gene Amp PCR System 9700 (Applied Biosystems, Foster City, CA, USA) for 34 cycles (30 s at 95 °C, 30 s at 60 °C, and 30 s at 72 °C). The resulting PCR products were digested using 2.5 units of restriction endonuclease (RSAI for rs2271715, BstNI for rs1878326, BANII for rs4945 and StyI HF for rs3743388, New England Biolabs Ipswich, MA, USA) per reaction, for 55 min at 37 °C. Kits for PCR reactions were supplied by Kapa Biosystems, (Kapa ready mix, Wilmington, DE, USA). PCR products and restriction fragments were visualized on a 3% agarose gel stained with ethidium bromide. The four SNPs after digestion yielded fragments as follows: for rs2271715 CC 287 bp, TT 231 bp and 56 bp, CT 287 bp, 231 bp and 56 bp, for rs1878326 AA 197 bp, CC 126 bp and 61 bp, AC 197 bp, 126 bp and 61 bp, for rs4945 CC 182 bp, AA 111 bp and 71 bp, CA 182 bp, 111 bp, and 71 bp, and for rs3743388 GG 313 bp, CC 231 bp and 82 bp, GC 313 bp, 231 bp and 82 bp.

## 4. Measurement of MFGE8 Serum Levels

MFGE8 protein levels were measured in sera derived from 70 SS, 76 SLE, 74 RA and 27 HC, using a commercially available ELISA kit (Human MFGE8 Elisa Kit, ABCAM, Waltham, MA, USA), according to the manufacturer’s instructions. All measurements were performed in duplicate, at 412 nm, using a plate reader (Versamax, Molecular Devices, San Jose, CA, USA).

## 5. Statistics

Allele and genotype frequencies in patients and HCs were determined for the four MFGE8 variants by SHEsis and SNPStats software. Genotype frequencies in control subjects for each SNP were tested for departure from the Hardy–Weinberg equilibrium. Genotype frequencies for all variants were compared in patients and HCs using the χ2 test and ORs and corresponding 95% CIs were estimated. Adjustment for the effects of age and gender was performed. Five genetic models (codominant, dominant, recessive, over dominant, and additive) were also determined.

We assessed two-group comparisons of continuous data, using t-tests or the Mann–Whitney test, when the data distribution was not normal. The SPSS v.26 and GraphPad Prism 8 software were used. We determined the correlation between gene expression data using a non-parametric Spearman’s test. A *p*-value of <0.05 was considered statistically significant.

## 6. Results

To investigate the allele and genotype distribution of MFGE8 gene variants rs2271715, rs1878326, rs4945 and rs3743388 among SS, RA, SLE patients, and HC, allele and genotype analysis was performed. While no significant differences were found in the allele and genotype distribution of the four MFGE8 studied variants, among SS and SLE patients, compared to HC after adjustment for age and sex, in the RA patient group, the CA genotype of rs4945 variant exhibited a protective effect against disease development (OR (95% CI): 0.58 (0.34–0.99), *p* = 0.043). No other significant associations were detected between the MFGE8 variants and any demographic, clinical, laboratory features or markers of subclinical atherosclerosis for any disease group. Of interest, following haplotype analysis, we found a significant protective effect of the CACG haplotype, in both RA (HC vs. RA: 0.11% vs. 0.04%, OR (95% CI): 0.26 (0.10–0.71), *p* = 0.009) and SS patients (HC vs. SS: 0.11% vs. 0.05%, OR (95% CI): 0.22 (0.06–0.8), *p* = 0.023) compared to HC (Table 1). However, this observation was not confirmed in the SLE population. 

Regarding MFGE8 serum levels, no significant differences were found between RA, SS, SLE and HC (Figure 1B). Similarly, after stratification of all disease cohorts and HC, according to the genotypes of the four MFGE8 variants, no differences in MFGE8 serum levels were detected (data not shown). Of interest, SS, but not RA or SLE, patients with IMT ≤ 0.9 displayed higher MGFE8 serum levels compared to those with >0.9 (mean ± SD: 594 ± 504 ng/μL vs. 468 ± 381 ng/μL, *p* = 0.03) (Figure 1C). No significant associations between markers of subclinical atherosclerosis and MFGE8 serum levels were detected in RA or SLE groups. 

## 7. Discussion

To our knowledge, this is the first time that the CACG MFGE8 haplotype was found to confer reduced risk for RA and SS development, with MFGE8 protein levels being reduced in SS patients with evidence of carotid arterial wall thickening. Previous studies conducted in Korean and Taiwanese SLE cohorts have reported that SLE patients carrying the MFGE8 rs2271715 and rs3743388 variants display higher MFGE8 serum levels [1] and increased risk for SLE compared to HC [1,7] (findings not confirmed in the present cohort). Additionally, we observed that the CA genotype of the rs4945 variant exhibits a protective effect against RA development, a finding that was not observed in SS and SLE cohorts. A recent study conducted in a Korean SLE cohort revealed that SLE patients carrying the A allele of the rs4945 variant a display higher erythrocyte sedimentation rate (ESR) compared to non-carriers [1]. However, no association with any clinical disease manifestation was observed in our disease cohorts. The apparently discrepant results between the different studies might reflect genetic differences between Caucasian and Asian patients. 

A limitation of our study is the relatively small number of patients enrolled in the study. Due to the study design, we retrospectively included only patients with available atherosclerosis-related data. Thus, these findings should be further explored in larger multicenter cohorts.

Taken together, we report a novel association of MFGE8 genetic variations in SS and RA susceptibility, along with reduced MFGE8 serum levels in SS patients with heightened atherosclerotic risk. These findings further strengthen the concept of shared pathophysiological mechanisms between atherogenesis and autoimmune diseases. 

## Figures and Tables

**Table 1:** The haplotype analysis of the MFGE8 gene variants rs2271715, rs1878326, rs4945 and rs3743388 in RA and SS patients compared to HC.

rs2271715	rs1878326	rs4945	rs3743388	HC (%)	RA (%)	SS (%)	† OR (95% CI) HC vs. RA	† OR (95%CI) HC vs. SS	*p*-Value HC vs. RA	*p*-Value HC vs. SS
C	A	C	G	0.11	0.04	0.05	0.26 (0.10–0.71)	0.22 (0.06–0.8)	0.009	0.023
T	A	A	G	0.19	0.16	0.16	0.51 (0.26–1.00)	0.43 (0.19–1.01)	0.052	0.053

† Adjusted for age and gender. Results are shown as haplotype frequency estimation. MFG-E8: milk fat globule epidermal growth factor 8; RA: Rheumatoid Arthritis; SS: Sjögren’s Syndrome HC: healthy controls; OR: Odds Ratio; 95% CI: 95% Confidence Interval; SNP: single nucleotide polymorphism; *p* ≤ 0.05.

## Data Availability

The data presented in this study are available on request from the corresponding author.
